# Inequalities and factors associated with adherence to diabetes self-care practices amongst patients at two public hospitals in Gauteng, South Africa

**DOI:** 10.1186/s12902-020-0492-y

**Published:** 2020-01-28

**Authors:** Chipo Mutyambizi, Milena Pavlova, Charles Hongoro, Wim Groot

**Affiliations:** 1 Research Use and Impact Assessment, Human Sciences Research Council, HSRC Building, 134 Pretorius Street, Pretoria, 0002 South Africa; 2 Department of Health Services Research, CAPHRI, Faculty of Health, Medicine and Life Sciences, Maastricht University, Maastricht University Medical Centre, Maastricht, The Netherlands

**Keywords:** Diabetes, Self-care behaviours, Inequalities, Determinants, Adherence, South Africa

## Abstract

**Background:**

Self- management is vital to the control of diabetes. This study aims to assess the diabetes self-care behaviours of patients attending two tertiary hospitals in Gauteng, South Africa. The study also seeks to estimate the inequalities in adherence to diabetes self-care practices and associated factors.

**Methods:**

A unique health-facilities based cross-sectional survey was conducted amongst diabetes patients in 2017. Our study sample included 396 people living with diabetes. Face-to-face interviews were conducted using a structured questionnaire. Diabetes self-management practices considered in this study are dietary diversity, medication adherence, physical activity, self-monitoring of blood-glucose, avoiding smoking and limited alcohol consumption. Concentration indices (CIs) were used to estimate inequalities in adherence to diabetes self-care practices. Multiple logistic regressions were fitted to determine factors associated with diabetes self-care practices.

**Results:**

Approximately 99% of the sample did not consume alcohol or consumed alcohol moderately, 92% adhered to self-monitoring of blood-glucose, 85% did not smoke tobacco, 67% adhered to their medication, 62% had a diverse diet and 9% adhered to physical activity. Self-care practices of dietary diversity (CI = 0.1512) and exercise (CI = 0.1067) were all concentrated amongst patients with higher socio-economic status as indicated by the positive CIs, whilst not smoking (CI = − 0.0994) was concentrated amongst those of lower socio-economic status as indicated by the negative CI. Dietary diversity was associated with being female, being retired and higher wealth index. Medication adherence was found to be associated with older age groups. Physical activity was found to be associated with tertiary education, being a student and those within higher wealth index. Self-monitoring of blood glucose was associated with being married. Not smoking was associated with being female and being retired.

**Conclusion:**

Adherence to exercising, dietary diversity and medication was found to be sub-optimal. Dietary diversity and exercise were more prevalent among patients with higher socio-economic status. Our findings suggest that efforts to improve self- management should focus on addressing socio-economic inequalities. It is critical to develop strategies that help those within low-socio-economic groups to adopt healthier diabetes self-care practices.

## Background

Diabetes Mellitus is a serious and common chronic illness globally, and a major cause of limb amputations, blindness, kidney failure and stroke [1]. It is reported that people living with diabetes are at an increased risk of developing additional health problems and infections when compared to people without diabetes [2]. The risk of cardiovascular diseases in people living with diabetes is double that for non-diabetics [3]. Furthermore, diabetes is associated with an excess risk of mortality from several non-vascular conditions such as cancer [4]. Diabetes and its complications are a major cause of mortality globally. The International Diabetes Federation (IDF) estimates that globally 463 million (9.3%) people had diabetes and that diabetes and its complications were the cause of over 4 million deaths amongst people aged 20–79 years old in 2019 [5]. South Africa is reported to have the highest prevalence of diabetes in the African region (12.7% in 2019) and the highest number of deaths due to diabetes among low and middle income countries in 2019 (89,800 deaths) [5].

The morbidity of diabetes is related to its diabetes-related complications and multimorbidity, which is associated with poor glycaemic control [6]. In South Africa, poor glycaemic control has been reported in hospital-based studies conducted across the country [7–11]. Furthermore, using the nationally representative South African National Health and Nutrition Examination Survey, Stokes et al. shows that among individuals with diabetes, 18.1% were treated but uncontrolled (had an HbA1c greater than or equal to 7%) [12]. According to the American Diabetes Association (ADA) and the Society for Endocrinology, Metabolism and Diabetes of South Africa (SEMDSA), a glycaemic level equal to or below 7% is considered optimum [6, 13]. Achieving this level of diabetes control is an outcome of a complex mix of both pharmacological and non-pharmacological management practices [1]. Thus, diabetes management requires actions by different role-players (such as patients, their families and health care providers) to ensure improved outcomes. Whilst pharmacological management consists of the use of medicines (oral hypoglycemics and or insulin therapy), non-pharmacological management involves person education and support in the adoption of diabetes self-care practices [1].

Based on international studies, the SEMDSA developed guidelines that are used for the management of diabetes in South Africa [13, 14]. As emphasised by the guidelines, adherence to diabetes self-care is an integral part of diabetes management, contributing to improved glycaemic levels, reduced development of diabetes complications and associated costs, and improved quality of life [6]. The essential components of diabetes self-care include for example healthy eating, physical activity, tobacco smoking cessation, weight management, medication adherence, self-monitoring of blood glucose levels, blood pressure and feet, routine screening of eye and renal complications [6, 15]. Adherence to these self-care practices is influenced by a variety of factors such as socio-economic status, diabetes education, health beliefs, education level, family history of diabetes and patient demographic characteristics [16–19].

A systematic review by Stephani et al. shows that the levels of adherence to diabetes self-care practices in Sub-Saharan Africa are poor and a threat to achieving improved health outcomes [20]. In South Africa, studies that have investigated patient management of diabetes have been limited to using qualitative methods to investigate the challenges people living with diabetes face in the management of the chronic illness [21–25]. Studies that used quantitative methods investigated the prevalence or distribution of dietary [10] and exercise practices amongst diabetics [26]. One study that assessed the factors associated with diabetes self-care practices focused on medication adherence only [27]. Our study expands on these previous studies and assesses adherence to diabetes self-care practices amongst diabetics visiting two tertiary hospitals in Gauteng, South Africa. A study of this nature is important for facilitating actions for improved diabetes self-care practices amongst people living with diabetes, particularly those from low resource settings, such as those visiting the public hospitals. Our study therefore aims to: (1) describe adherence to the following diabetes self-care behaviours: dietary diversity, medication adherence, physical activity, self-monitoring of blood-glucose, avoiding smoking and limited alcohol consumption; (2) estimate the inequalities in these self-care behaviours using Concentration Indices (CIs); (3) describe the association between adherence to diabetes self-care behaviours and patients’ demographic characteristics.

## Methods

### Study setting

The study was conducted in the Tshwane health district, which is one of the five districts within the Gauteng province. Tshwane is the third most populous district within the province, has an unemployment rate of 21.1%, a Gini coefficient of 0.64, and a medical insurance coverage of 30.5% [28, 29]. The majority of the population within the district is African (78%). Health care is provided via private and public health care facilities. Public health care is provided via a hierarchical referral health care system consisting of clinics, community health care centres and hospitals.

Data for the study was collected at two tertiary hospitals. Both hospitals operate diabetes clinics that serve similar catchment populations and are accessible to the district urban population and other outlying areas. Patients referred to the clinics, usually have diabetic complications or poorly controlled blood sugar. The diabetes clinics at the hospitals are open on specific clinic days during the week and operate on a structured consultation schedule. Health education at the hospitals is provided by a health education team consisting of the nurses, medical doctors and dieticians. Patients are scheduled to attend the clinic every 3 months and at each consultation, a different focus is set such as foot examination, eye examination or dietician consultation.

The study was conducted alongside our previously published paper on catastrophic health expenditure and impoverishment amongst people living with diabetes [30]. Additional information on diabetes related issues and health behaviours was also collected.

### Sample size

The single population proportion formula was used to estimate the study sample size. Using a confidence interval of 95%, an absolute error of 0.05 and 50% proportion, the sample size was estimated at 385. To account for the possibility of refusals, we added 115 patients to this estimated sample size. A total of 503 patients were invited to participate in the study.

### Data collection

Data collection was conducted in March to April and November to December of 2017. All patients visiting the hospital during the data collection period, were invited to participate in the study. Patients who were severely ill and could not communicate, were excluded from the survey. Face to face interviews were conducted by four experienced research assistants, trained on the study protocol and data collection procedures. For this study questionnaire development was guided by the South African National Health and Nutrition Examination Survey data collection tool and adapted to the South African public hospital context. The SANHANES-1 assessed non-communicable diseases in South Africa as well as the health and nutrition status of the South African population. The questionnaire is easy to administer and practical to use with illiterate populations and the elderly. The questionnaire used for this study consists of a food frequency questionnaire, the Global Physical Activity Questionnaire (QPAQ), questions related to alcohol, tobacco use, self-monitoring of blood glucose and the Morisky medication adherence instrument. The questionnaire was pretested with 8 patients at one of the hospitals to ensure validity and reliability. Amendments were then made were necessary. Given that the primary objective of the study was to collect information related to household expenditure and income, data collection was restricted to individuals above the age of 21 years. No incentives or inducement was offered to participate in the study. Quality checks of all completed questionnaires was done by the data collection supervisor at the end of each data collection day.

### Ethical approval

Ethical approval for data collection was obtained from the Research Ethics Committee of the Human Sciences Research Council (HSRC) (ref: 14/23/11/16) and the University of Pretoria Research Ethics Committee (Protocol number 114/2017). Each participant provided written informed consent and clinical managers were informed of the study.

### Inequalities in diabetes self-care behaviours

To determine the inequalities in diabetes self-care behaviours, our study makes use of the widely employed Concentration Index (CI). The CI takes on a value of 0 when there are no inequalities in the outcome variable, a negative value when the outcome variable is more concentrated amongst the poor and a positive value when the outcome variable is more concentration amongst the rich. It is measured as twice the covariance of the outcome variable and living standards variable all divided by the mean of the outcome variable [31].

1 $$ CI=\frac{2}{\mu}\mathit{\operatorname{cov}}\left(h,r\right) $$

Our study makes use of the Erreygers corrected CI, where μ is the mean of the variable, CI is the standard CI, b is the maximum value of the variable (in this case 1) and a is the minimum value of the variable (in this case 0). Our study makes use of STATA’s *conindex* command [32].

2 $$ E(h)=\frac{4\mu }{b-a} CI $$

In this study, to determine the wealth categories, we make use of the wealth index calculated via multiple correspondence analysis. A set of 10 household assets and living standard measures were used in the estimation of the wealth index. The list of items is as follows: housing type, water and sanitation services, ownership of a television, refrigerator, 4 plate stove, radio, cell phone, computer and car. The wealth index was used to estimate the CIs and also, to categorise the respondents into wealth quintiles.

### Study variables and analysis

#### Dietary diversity

A healthy diet is an essential part of diabetes management. Because one single food cannot be the source of all required nutrients, consuming a varied diet increases the likelihood of consuming all required nutrients [33]. Dietary diversity scores are an indication of diet nutrient adequacy [34]. Therefore, to assess the diet quality amongst the study participants, our study makes use of the Dietary Diversity Score (DDS). DDS is defined as the number of food groups consumed by an individual over a 24 h period [35]. Survey respondents were asked to recall the foods they ate the day before the interview. These food items were then linked to corresponding food groups and these groups were used to calculate the DDS. Our study followed methods developed in other studies in the estimation of DDSs [36, 37]. The nine food groups were: cereals, roots and tubers; vitamin-A-rich vegetables and fruits; other fruit; other vegetables; meat, poultry and fish; legumes; fats and oils; dairy products and eggs. A nine-point scale was then created using the number of food groups consumed by the individual. Dietary inadequacies are associated with a DDS below 4 [34, 36, 37]. Consistent with other studies, the operational definition for DDS adherence in this study is respondents who have a DDS greater than or equal to 4 [33]. Thus, a binary variable was created that took a value of one when DDS was > = 4 and otherwise, it took a value of zero.

#### Medication

The level of medication adherence was measured using the Morisky’s instrument – a validated four-question preformed questionnaire [38]. Consistent with other studies that have applied the Morisky’s instrument, individuals were considered adherent to medication if they gave negative responses to all four questions [39]. Medication adherence was included as a binary variable which took on a value of one if patients adhered to medication and otherwise, it took a value of zero.

#### Physical activity

The World Health Organisation recommends at least 150 min of moderate intensity physical activity and 75 min of vigorous intensity physical activity per week [40]. In our study, individuals were considered adherent to physical activity if they reported taking part in more than 2 hr of any of the following physical activities per week: bicycling, brisk walking/jogging, sport activities, strength exercises, aerobic exercises and any other exercises.

#### Self-monitoring of blood-glucose

Although self-monitoring of blood-glucose is recommended for diabetics, the International Diabetes Federation (IDF) and SEMDSA advise that the frequency of self-monitoring of blood-glucose is dependent on individual clinical needs [13, 41]. The SEMDSA guidelines recommend those using insulin to test at least once a day and for those using oral medication testing 3–5 times per week may be sufficient. Our operational definition for adherence to self-monitoring of blood-glucose is conducting a glucose test everyday over the past week for patients on insulin and at least 3 times per week when using tablets [13].

#### Smoking status

In this study, individuals were asked to report if they currently smoked tobacco. The operational definition for non-smoking-related adherence was a respondent who reported not currently smoking tobacco. A binary variable was created for non-smoking-related adherence which took on a value of 1 when individuals did not smoke and otherwise it took on a value of zero.

#### Alcohol consumption

Individuals were asked to report how often they consumed alcohol in the last 12 months. This variable was included as a binary variable that took on a value of one if the individual reported never consuming alcohol or consuming alcohol up to four times a month and a value of zero when individuals reported consuming alcohol more than 4 times a week.

#### Socio-demographic characteristics

Our study also included the following variables: age category (21–40 years, 41–60 years and 60+ years), sex, race (African/non-African), marital status (single, married, divorced, widowed and co-habiting), having children (yes/no), education level (primary, secondary, tertiary), employment status (unemployed, formally employed, informally employed, student and retired), household size (1–4 members/5+ members) and wealth index quintile (quintile 1, quintile 2, quintile 3, quintile 4 and quintile 5).

Statistical analysis was conducted in STATA 13. First descriptive analysis was performed. Then, multivariate Logistic regression analysis was conducted to assess the factors associated with each of the diabetes self-care behaviours. Thus, we have separate regressions for each self-care behaviour.

## Results

Out of the 503 patients who were invited to take part in the survey, 405 patients agreed to be interviewed. Due to incomplete data, 9 patients were excluded from the analysis. Thus our study sample included 396 diabetes patients.

### Descriptive statistics

Table 1 shows the socio-demographic characteristics of the study sample. The majority of our study sample was between the age of 41 and 60 years. Approximately 61% were female, 76% were African, 35% were single, 86% reported having children, 66% had secondary education, 49% were unemployed and 64% came from households with 1 to 4 members.

**Table 1:** Socio-demographic characteristics of diabetic patients (*N* = 396)

Variable	Frequency	Percent
Age category		
21–40	98	25.39
41–60	171	44.30
60+	117	30.31
Sex		
Male	155	39.24
Female	240	60.76
Race		
African	300	76.34
Non-African	93	23.66
Marital status		
Single	136	35%
Married	162	41%
Divorced	36	9%
Widowed	52	13%
Co-habiting	7	2%
Children		
Yes	337	85.97
No	55	14.03
Education		
Primary	65	16.75
Secondary	255	65.72
Tertiary	68	17.53
Employment status		
Unemployed	184	49%
Formal	107	29%
Informal	30	8%
Student	8	2%
Retired	44	12%
Household size		
1–4	249	63.68
5+	142	36.32

*Std.dev* Standard Deviation

#### Diabetes self-care behaviours amongst people living with diabetes

Table 2 shows the distribution of diabetes self-care practices within our study sample. The majority of the participants were adherent to limited alcohol consumption (98.72%). Approximately 92.18% of the study sample was found to be adherent to self-monitoring of blood-glucose. In addition to the results in Table 2, the majority of the study participants reported having a machine to monitor blood glucose (92%). Of those who did not adhere to self-monitoring of blood-glucose, 8% reported not having a machine to measure blood glucose. Table 2 also shows that non-smoking adherence was also common at 85.3%. Approximately 66.84% adhered to their diabetes medication regime and 62.27% had dietary diversity. Over 90% of the study sample did not adhere to physical activity. Being too sick to exercise was the most commonly reported reason for non-adherence to exercise.

**Table 2:** Diabetes self-care behaviours amongst diabetic patients

Variable	Frequency	Percent
Limited alcohol consumption (*N* = 391)		
No	5	1.28
Yes	386	98.72
SMBG (*N* = 358)		
No	28	7.82
Yes	330	92.18
Non-smoking (*N* = 381)		
No	56	14.7
Yes	325	85.3
Medication Adherence (*N* = 374)		
No	124	33.16
Yes	250	66.84
Dietary diversity (*N* = 387)		
No	146	37.73
Yes	241	62.27
Exercise (*N* = 396)		
No	359	90.66
Yes	37	9.34

Table 3 shows the distribution of self-care behaviour by wealth index quintile. Adherence to exercise and self-monitoring of blood glucose appeared to increase by wealth quintile whilst adherence to non-smoking decreased by wealth quintile. The majority of individuals who adhered to dietary diversity, belonged to the fifth quintile. In all wealth quintiles, the majority of individuals adhered to their diabetic medication and limited alcohol consumption. In addition, this study found that a majority of the study participants adhered to three out of the six diabetes self-care behaviours (42%). This was followed by those who adhered to four out of the six diabetes health care behaviours (34%). Approximately 18% adhered to two out of the six diabetes self-care behaviours, 4% adhered to five and 1% adhered to none.

**Table 3:** Distribution of self-care behaviour by wealth quintile

Variable	Wealth Index quintiles (%)					
	1	2	3	4	5	Overall
Dietary diversity	47%	65%	67%	64%	71%	62%
Medication adherence	74%	72%	57%	63%	67%	67%
Exercise	4%	7%	11%	11%	17%	9%
SMBG	90%	92%	92%	93%	95%	92%
Non-Smoking	91%	88%	83%	83%	81%	85%
Limited alcohol consumption	97%	99%	99%	100%	98%	99%

#### Inequalities in diabetes self-care behaviours

Table 4 shows the CIs for diabetes self-care behaviours, namely dietary diversity, medication adherence, physical activity, self-monitoring of blood-glucose and non-smoking status. Due to the small sample sizes and loss of statistical power, we do not present results for inequalities in limited alcohol consumption. Whilst the CIs for dietary diversity, physical exercise and non-smoking were statistically significant, the CIs for medication adherence and self-monitoring of blood-glucose were all statistically insignificant. From the table, dietary diversity and physical exercise were all concentrated amongst the rich participants whilst non-smoking was concentrated amongst the poor participants.

**Table 4:** Inequalities in diabetes self-care behaviours

Variable	Obs	Index	95% Confidence interval		SE	*P*-value
Dietary diversity	387	0.1512	0.0405	0.2619	0.0565	0.0077
Medication adherence	374	−0.0909	−0.2009	0.0190	0.0561	0.1059
Exercise	396	0.1067	0.0413	0.1722	0.0334	0.0015
SMBG	358	0.0275	−0.0368	0.0918	0.0328	0.4019
Non-Smoking	381	−0.0994	−0.1810	− 0.0178	0.0416	0.0175

*Obs* Observations, *SE* Standard Error, *CI* Concentration Index

#### Factors associated with diabetes self-care behaviours

The results of the five separate multivariate logistic regressions for each of the self-care behaviours, namely dietary diversity, medication adherence, physical activity, self-monitoring of blood-glucose and non-smoking status are presented in Table 5. Below, the key findings are summarized.

**Table 5:** Factors associated with diabetes self-care behaviours (multivariate logistic regressions)

Variable	Dietary diversity		Medication adherence		Exercise		SMBG		Non-Smoking	
	OR	SE	OR	SE	OR	SE	OR	SE	OR	SE
Age 41–60	0.8098	0.2650	2.6326***	0.9112	1.0012	0.5289	0.6822	0.4510	1.4149	0.6725
Age 60+	0.7575	0.3343	6.3777***	3.2640	0.3587	0.3091	0.2692*	0.2274	0.7221	0.4675
Female	1.6065*	0.4134	1.1396	0.3174	1.0393	0.4417	0.4152	0.2463	7.6939***	3.1335
Non-African	0.8134	0.2589	0.3551***	0.1206	0.5158	0.2743	0.2511**	0.1499	0.2684***	0.1130
Married	1.1395	0.3493	1.1414	0.3842	1.0732	0.5521	2.9344*	1.8681	1.5144	0.7080
Divorced	1.2395	0.5959	0.8302	0.4247	0.7673	0.6072	5.2781	5.9890	0.7425	0.4607
Widowed	0.7827	0.3494	0.3490**	0.1705	0.8535	0.7111	1.2193	0.8426	2.1414	1.7505
Co-habiting	1.3038	1.1209	0.7091	0.6763	1.0559	1.3268	1.0000	(empty)	1.2239	1.4795
No Children	0.7534	0.2888	0.7997	0.3158	0.7393	0.4442	0.5485	0.3869	1.2525	0.6677
Secondary education	1.4419	0.4926	0.8931	0.3604	4.4887	4.7725	0.6377	0.4694	0.4430	0.3025
Tertiary education	1.3933	0.6451	0.9553	0.4877	6.3922*	7.1806	0.3830	0.3572	0.4116	0.3236
Formal employment	1.1233	0.3388	0.8614	0.2774	2.1005	0.9704	0.9272	0.5893	1.8184	0.8490
Informal employment	0.8938	0.4177	0.9514	0.4837	1.0000	(empty)	1.3920	1.6196	0.7059	0.4361
Student	1.0663	0.9883	0.7519	0.6576	6.0037*	5.6769	1.0000	(empty)	1.0000	(empty)
Retired	2.3336*	1.1345	1.3734	0.7383	3.2434	2.5307	0.4892	0.3454	4.2325*	3.2636
Household size 5+	1.2387	0.3253	0.8263	0.2378	0.4363*	0.2036	0.4860	0.2506	1.2194	0.4990
Wealth Quintile 2	2.8709***	1.0212	1.0724	0.4295	1.6891	1.2799	2.9264	2.0445	0.9778	0.5778
Wealth Quintile 3	2.4113***	0.8814	0.3400***	0.1436	2.8607	2.1307	2.1131	1.4218	0.5372	0.3121
Wealth Quintile 4	2.4690**	0.9726	0.4972	0.2178	3.6635*	2.7972	4.0168	3.4623	1.1018	0.6825
Wealth Quintile 5	4.6455***	2.1532	0.9902	0.4739	5.1521**	4.1241	4.1036	3.6775	0.7928	0.5055
Sample (n)	334		324		316		298		322	
Pseudo R^2^	0.0604		0.1141		0.1156		0.1471		0.2189	

Reference categories: Age 21–40 years, male, African, single, having children, primary education, unemployed, household size 1–4 and wealth quintile 1 (lowest wealth). All outcome variable are binary in which a value of 1 indicates a diverse diet, medication adherence, exercise, self-monitoring of blood glucose and smoking

*OR* Odds Ratio, *SE* Standard Error

##### Dietary diversity

The variables significantly associated with dietary diversity were being female (Odds Ratio [OR] 1.60; Standard error [SE] 0.41) versus being male, retired (2.33; 1.13) versus unemployed, wealth quintile 2 (2.87; 1.01), wealth quintile 3 (2.41; 0.88), wealth quintile 4 (2.46; 0.97) and wealth quintile 5 (4.65; 2.15) versus wealth quintile 1 (lowest wealth).

##### Medication adherence

The variables significantly associated with medication adherence were age category 41–60 years (2.63; 0.91), age category 60+ years (6.37;3.26) versus age category of 21–40 years, being non-African (0.35; 0.12) versus being African, widowed (0.34; 0.17) versus being single, wealth quintile 2 (0.34; 0.14) versus wealth quintile 1.

##### Physical exercise

The variables significantly associated with physical exercise were tertiary education (6.39; 7.18) versus primary education, being a student (6.00; 5.67) versus being unemployed, household size > 5 (0.43; 0.20) versus household size < 5, wealth quintile 4 (3.66; 2.79) and wealth quintile 5 (5.15; 4.12) versus wealth quintile 1.

##### Self-monitoring of blood-glucose

The variables significantly associated with self-monitoring of blood-glucose were age category of 61+ years (0.26; 0.22) versus age category of 21–40 years, and being non-African (0.25; 0.14) versus being African, being married (2.93; 1.86) versus being single.

##### Non-smoking

The variables significantly associated with not smoking were being female (7.69; 3.13) versus being male and being non-African (0.26; 0.11) versus being African, being retired (4.23; 3.26) versus being unemployed. Thus, female, African or retired people living with diabetes in our study had higher odds to adhere to non-smoking compared to male, non-African or unemployed respondents whose odds to adhere to non-smoking were lower.

## Discussion

Diabetes is a serious chronic illness that leads to the development of complications and early mortality if not controlled and managed. In this paper, we examined diabetes self-care management practices of patients attending two tertiary hospitals in Gauteng, South Africa. Our analysis focused on six self-care practices of dietary diversity, medication adherence, physical activity, self-monitoring of blood-glucose, non-smoking status and limited alcohol consumption. An assessment of adherence to diabetes self-care practices and their inequalities together with the identification of factors associated with these behaviours, is important for the design of strategies to control diabetes. Our findings for each diabetes self-care practice are discussed below.

Although a variety of dietary approaches (such as low carbohydrate diets or low fat diets) have been applied in the management of diabetes, there has not been one single diet that has been identified as being superior to the rest in diabetes management [13]. However, it is acknowledged that a diet with varied nutrients is associated with improved diabetes management outcomes [13]. A diet that lacks diversity is often an indication of food scarcity, which is often associated with malnutrition [33]. Our hospital-based study revealed that approximately 38% of our study sample had poor dietary diversity (DDS < 4). Using a nationally representative South African dataset, a study by Labadarios et al. showed that at the national level, approximately 38% have poor dietary diversity [33]. Our findings are lower than those reported among Ethiopian type 2 diabetes patients in a hospital-based study which found that 76% of patients did not adhere to recommended diet [42]. They are also lower than findings from a South African hospital-based study by Okonta et al. who finds that 99% of their sample did not follow any diet [26]. With regards to inequality in DDS, our findings show that having a diverse diet is concentrated amongst the better off, as indicated by the positive concentration indices. This finding is also evident from the regression results which show that being within a highest wealth quintile is associated with diverse diets. This finding corroborates the findings from a study by Tiew et al. who make use of a different measure of DDS and finds that in their type 2 diabetes sample, higher income is associated with having a diversified diet [43].

Self-monitoring of blood glucose is an essential component of diabetes self-care and prevention of hypoglycaemia [6] as it guides decision making regarding adjustments in medication dosages, exercise regimes and dietary intake. By self-monitoring glucose levels participants become actively involved in achieving targeted glycaemic levels. Using a randomised prospective study of 689 type 2 diabetes patients Guerci et al. show that the group who engaged in self-monitoring of diabetes had lower HbA1c levels when compared to the control group [44]. The benefits of SMBG in type 1 diabetes has also been demonstrated elsewhere [45]. Approximately 92% of our hospital-based study sample practices self-monitoring of blood glucose levels. This is considerably higher than those reported among type 2 diabetes patients visiting a hospital in Ethiopia in which approximately 84% did not adhere to self-monitoring of blood glucose [42]. Our findings are much higher than an Indian based study that cited unaffordability of glucometers as the reason for poor testing [46], and also higher than findings from a sample of Chinese Americans in which 27% monitored their blood glucose daily [47]. These two studies are however not hospital-based studies. The high levels recorded in our study are perhaps due to the fact that most patients reported having a machine to self-test blood glucose. Consistent with a study by Harris et al. on a United States population, we found no association between socio-economic status and self-monitoring of blood glucose amongst diabetics within a national health interview survey [48].

Non-adherence to diabetes medication is associated with uncontrolled diabetes and an accelerated development of diabetes complications such as retinopathy, nephropathy and neuropathy. Adherence to diabetic medication has been previously investigated [16, 39, 49, 50]. A systematic review by Krass et al. reported a wide range of diabetes medication adherence (between 38.5 and 93.1%) which mostly varied as a result of the method used to measure adherence [49]. Our study shows that 67% of our participants were adherent to diabetes medication. Although our study makes use of the Morisky instrument to measure adherence our finding is consistent with results from a type 2 diabetes hospital-based study in Limpopo, South Africa that used self-reported data recall of taking medication and found 70% of the participants adhered to treatment [27]. Our results showed that older age was associated with adherence to diabetic medication whilst being non-African compared to being African was associated with none adherence to diabetic medication.

Increased physical activity is associated with reduction in HbA1c levels [51]. Our study finds that over 90% of our sample did not take part in physical activity of at least 2 h per week. This finding is higher than findings from a sample of Chinese Americans (who lived in Ohio and Chicago) in which 60% exercised less than 5 d per week [47]. Our findings are however consistent with a Mamelodi hospital-based study in South Africa by Okonta et al. who found that 92% of the sample did not exercise regularly [26]. Our findings are also consistent with findings reported from a study on type 2 diabetic patients living in underserved communities in New York, which found that physical activity was the diabetes self-care behaviour with the lowest rates of adherence [52]. Physical activity was more concentrated amongst those within the higher wealth quintiles. This finding is also supported by findings from the regression which also show that those within higher wealth quintiles were more likely to adhere to physical exercise. Consistent with a hospital-based study in Ghana [17], and a study on Chinese Americans with type 2 diabetes [47], we also find that higher education is associated with adherence to exercise.

Our findings show that approximately 15% of the study participants were smokers. This finding is consistent with findings from a study at Baragwanath Hospital, South Africa which found a self-reported smoking prevalence amongst diabetics of 16% [53] and a study from India which found that approximately 14% of the sample reported smoking in the previous week [46]. Using CIs our study shows that those within lower socio-economic groups were more likely to adhere to non-smoking although we did not find this relation in the regression analysis. Consistent with findings from a study in India our study also finds that not smoking is associated with gender, with females more likely to not smoke when compared to males [46].

Diabetes self-care practices also involve the avoidance of harmful alcohol consumption [1, 54]. The IDF recommends a maximum intake of two standard drinks per day [15]. Approximately 99% of the sample abstained from alcohol consumption. This finding is higher than that recorded by a study at public health care facilities in the North West Province, South Africa that found that 65% of diabetics abstained from alcohol consumption [55]. It is possible that patients quit or reduce their alcohol consumption after having been diagnosed with diabetes.

Whilst diabetes self-care is mostly the patient’s responsibility [56], it is well established that health care professionals play a role in supporting diabetes self-care by patients and ultimately improve clinical outcomes. The role of health care professionals in relation to diabetes self-care is gaining an increasing attention in the literature [57]. To encourage adherence to self-care, there is a call for health care professional and patient interactions to be collaborative rather than directive [58]. Such interactions encourage patient involvement, via the identification of problems in diabetes management [59]. The approach promotes shared decision making and allows for the building of a good relationship between the patient and health care professional [58]. In clinical practice, this will enable health care professionals and patients to make and agree on health care choices together.

### Limitations and implications for future research

Our study has some limitations. Diabetes foot care is an integral part of diabetes management. People living with diabetes are encouraged to examine their feet on a regular basis and examine the insides of their shoes before putting them on. Our study however did not collect data on patient foot examination. The hospital-based respondent selection may have missed the inclusion of those diabetics who are not seeking care. The degree of self-care among that group is not studied. The findings from this study may also be limited by the possibility of social desirability bias during the face-to-face interviews. Furthermore, the use of a cross sectional dataset limits any casual interpretations. The findings from our study are based on data collected from two hospitals in Gauteng and thus may not be applicable to the whole of South Africa. However, we believe that the study provides insights into the self-care behaviours of diabetics in patients visiting the two South African public hospitals. Future research should attempt to investigate diabetes self-care management practices of patients attending private health care facilities in South Africa, in particular qualitative studies that explore the factors influencing diabetes self-care. Furthermore, future research should look into the role of family and friends in the adoption of healthy lifestyle and also focus on the barriers to the adoption of diabetes self-care practices.

## Conclusion

This study provided findings on the diabetes self- management practices of patients attending two tertiary hospitals in Gauteng, South Africa and their association with demographic variables. The study showed variation in adherence to diabetes self-care practices amongst the diabetes patients. Whilst high levels of adherence were reported in some self-care behaviours, our findings show that the extent to which patients adhere to the diabetes self-care behaviours of exercising, dietary diversity and medication are low and might have negative implications for diabetes health outcomes. The CIs showed that dietary diversity and exercise were associated with higher socio-economic status whilst non-smoking was associated with low socio-economic status. A study of this nature is important for health care professionals, in particular health care professionals dealing with economically disadvantaged patients. It is critical for strategies to be developed that help different-socio-economic groups to adopt healthier diabetes self-care practices. Future studies on this topic should also include individuals with diabetes who do not seek care to be able to draw generalized conclusions.

## Data Availability

The dataset used in this study is available upon request from the HSRC.

## Abbreviations

ADA: American Diabetes Association
CI: Concentration Index
DDS: Dietary Diversity Score
HSRC: Human Sciences Research Council
IDF: International Diabetes Federation
SEMDSA: Society for Endocrinology, Metabolism and Diabetes of South Africa
